# Chlamy_ChloroPred: a deep learning-based, highly accurate binary classifier for chloroplast protein prediction in the model microalga, *Chlamydomonas reinhardtii*, with potential cross-proteome versatility

**DOI:** 10.3389/fmicb.2026.1744805

**Published:** 2026-02-23

**Authors:** Hong Il Choi, Sung Ho Lee, Il Hyung Lee, Yong Jae Lee, Jin-Ho Yun, Dong-Yun Choi, Dae-Hyun Cho, Bum-Soo Shin, Junyoung Chun, Dong Won Lee, Hee-Sik Kim

**Affiliations:** 1 Cell Factory Research Center, Korea Research Institute of Bioscience and Biotechnology (KRIBB), Daejeon, Republic of Korea; 2 Department of Bioresource and Environmental Engineering, University of Science and Technology (UST), Daejeon, Republic of Korea; 3 Daemyung Vision Co., Ltd., Yongin-si, Republic of Korea; 4 Department of Integrative Biotechnology, Sungkyunkwan University, Suwon-si, Republic of Korea

**Keywords:** *Chlamydomonas reinhardtii*, chloroplast protein, deep learning, microalgae, neural network, protein localization prediction

## Abstract

**Introduction:**

The chloroplast, a living relic of an ancient endosymbiotic interaction between a microalga and a microbe and the principal subcellular organelle responsible for biological CO_2_ assimilation, is emerging as a key target for research to enhance photosynthetic efficiency beyond its current limitations. Given that accurate protein localization is a prerequisite for the in-depth scientific investigation and practical application of the membrane-compartmentalized photosynthetic organelle, numerous computational prediction tools have been proposed, yet their accuracy remains unsatisfactory.

**Methods:**

To address the limitation, we herein present Chlamy_ChloroPred, a newly developed deep learning-based framework composed of multi-layered artificial neural networks, carefully designed to perform binary classification of chloroplast proteins in the model photosynthetic microorganism, *Chlamydomonas reinhardtii*. The model captures locality-aware features of determinant amino acid residues in the chloroplast transit peptide (cTP), generally located within the ~50-amino-acid N-terminal region of mature chloroplast proteins, through the integration of ProtBERT-BFD embeddings, stacked bidirectional long short-term memory (BiLSTM) networks, and an attentive pooling layer.

**Results and discussion:**

Our model achieved an accuracy of 0.8462 for the *C. reinhardtii* proteome, outperforming widely used localization predictors, including TargetP 1.1 (0.4970), TargetP 2.0 (0.7396), and PredAlgo (0.7738) under a binary classification scheme. Comparative analyses further demonstrated that Chlamy_ChloroPred exhibits competitive performance relative to the current state-of-the-art model, PB-Chlamy (0.8521), under identical evaluation conditions. Notably, despite being trained solely on the algal proteome, Chlamy_ChloroPred showed substantial cross-species versatility when applied to the proteome of the terrestrial plant, *Arabidopsis thaliana*, achieving an accuracy of 0.7316 – representing a 12.6% improvement over TargetP 2.0, a predictor with previously demonstrated cross-proteome versatility. This likely stems from the model’s robust ability to capture conserved features of chloroplast proteins across proteomes from diverse photosynthetic lineages.

**Conclusion:**

We developed a deep learning–based framework, Chlamy_ChloroPred, that integrates carefully designed neural layers with low computational complexity, achieving high predictive accuracy and interpretability. We believe that Chlamy_ChloroPred represents a compelling alternative to existing predictors, especially when accurate inference of chloroplast proteins is required.

## 1 Introduction

There is a nagging concern about the climate crisis caused by exploding anthropogenic CO_2_ emissions. Microalgae, photosynthetic microeukaryotes, are emerging as a promising biological platform to combat the global threat due to their ability to devour CO_2_ while converting it into valuable biocompounds (Choi et al., 2021). In the photosynthetic cells, biological CO_2_ fixation occurs in the chloroplast—like other terrestrial plants, a subcellular compartment that contains various photosynthesis-related proteinaceous machineries. In addition, the organelle plays a pivotal role in starch, amino acid, and lipid metabolism, all of which are essential for survival of the photosynthetic organisms (Wang et al., 2023). Since the complex, cascading biochemical reactions occur through the combinatorial functions of groups of enzymes co-localized within the chloroplast, accurately identifying the localization of proteins is one of the most important prerequisites for thorough understanding of life processes and their applications from scientific and engineering perspectives.

Although the chloroplast contains its own bacteria-like circular genome and transcription/translation apparatus—evidence of evolutionary endosymbiotic interaction between a microalga and a microbe that has become another intriguing characteristic of the chloroplast, attracting considerable scholarly interest (Dyo and Purton, 2018; Kang et al., 2021), most of its proteins are encoded by nuclear DNA. This spatial discordance necessitates sophisticated delivery mechanisms to properly localize and ensure the functionality of the nuclear-encoded proteins in the chloroplast (Leister, 2003; Teufel et al., 2022; Wang et al., 2023). Translocation of a chloroplast protein (CP) into the chloroplast is initiated by the recognition of the chloroplast transit peptide (cTP), which is an extension sequence that covalently fused to the N-terminal region of the premature CP. After the CP is imported *via* the TOC-TIC (translocon at the outer chloroplast membrane—translocon at the inner chloroplast membrane) supercomplex, the cTP is ultimately cleaved by a stromal processing peptidase upon its successful arrival inside the target organelle, leaving behind a folded mature protein (Lee and Hwang, 2018). Given the import process, cTP acts as a crucial biological label that directs the cargo (i.e., CP) to the chloroplast *in vivo*, suggesting that its presence may be a reliable indicator of a protein’s final subcellular destination (Caspari, 2022).

Fueled by the remarkable progress in artificial intelligence (AI), a number of amino acid sequence-based protein localization prediction tools have been suggested (Tardif et al., 2012; Almagro Armenteros et al., 2019; Teufel et al., 2022; Wang et al., 2023). Such *in silico* prediction programs provide good alternatives to reliable-yet-arduous conventional localization methods, such as fluorescence tagging and immunohistochemistry (Bernstein et al., 1994; Kong et al., 2015; Mackinder et al., 2017; Caspari, 2022; Wang et al., 2023), due to its immediacy and throughput. As interest in the photosynthetic organelle has increased, several predictors have also been proposed that are specifically designed to classify chloroplast proteins, which focus on the *N*-terminally located potential cTP region (Schein, 2001; Emanuelsson et al., 2008). However, they suffer from low predictive effectiveness, which can be attributed to several systematic reasons, including: (i) cTPs share loosely conserved properties in terms of amino acid length and composition—despite the identification of a handful of canonical consensus motifs (e.g., V/I-X-A/C), which precludes readily deciphering the common denominators of CPs, ultimately impeding the construction of a precise forecaster (Bruce, 2001; Franzén et al., 2001; Gavel and von Heijne, 2001; Tardif et al., 2012); (ii) the model architectures employed in previous programs may retain insufficient capability to figure out the enigmatic feature of cTPs; and (iii) the limited quantity and suboptimal quality of the input data used during training process might also adversely affect the model’s performance.

In this study, we demonstrate a novel binary classifier for chloroplast proteins, which is specially established for the green microalga, *Chlamydomonas reinhardtii*—an ideal model photosynthetic organism for biotechnological studies and cell factory development that awaits a tailored CP predictor to expedite the chloroplast research workflow (Tardif et al., 2012; Dyo and Purton, 2018; Surridge, 2022; Wang et al., 2023; Einhaus et al., 2024). To address the problematic issues raised in previous cases, we constructed and trained a multi-layered deep learning framework, named Chlamy_ChloroPred, which consists of a sequential connection of the following: a feature extractor based on ProtBERT-BFD that is a Transformer-based protein language model pre-trained based on 2.5 billion protein sequences (Elnaggar et al., 2022; Wang et al., 2023); a triply stacked RNN-BiLSTM (recurrent neural network-bidirectional long short-term memory) layer; a 4-head multi-head attention module; and an attentive pooling network. These components were brought together to unravel the barely explicit properties of cTPs. Furthermore, the training used hundreds of up-to-date, experimentally proven input data collated from various *C. reinhardtii* proteomics literature sources, considering the significance of data quality and quantity in creating an accurate predictive model. As a result, we successfully developed a binary classifier for CP with an accuracy of 0.8462 for the test dataset, representing a 14.41% improvement over the widely accepted benchmark program, TargetP 2.0 (accuracy of 0.7396) (Almagro Armenteros et al., 2019), when evaluated for the same dataset. Comparative analyses further demonstrated that Chlamy_ChloroPred outperforms broader predictors, such as TargetP 1.1—the predecessor of TargetP 2.0 (accuracy of 0.4970)—and PredAlgo (accuracy of 0.7738) (Tardif et al., 2012), and exhibits competitive performance relative to the current state-of-the-art (SOTA) model, PB-Chlamy (accuracy of 0.8521) (Wang et al., 2023), under a binary classification setting. Although our program was trained solely on *C. reinhardtii* proteomics, it also achieved higher accuracy (0.7316) than the cross-proteome benchmark, TargetP 2.0 (0.6500), when tested on CP data from another photosynthetic model, *Arabidopsis thaliana*. On balance, we believe that the Chlamy_ChloroPred program, with its high predictive performance and versatility, will serve as a breakthrough tool in future chloroplast and photosynthesis studies.

## 2 Materials and methods

### 2.1 Input data acquisition

**Figure 1:**
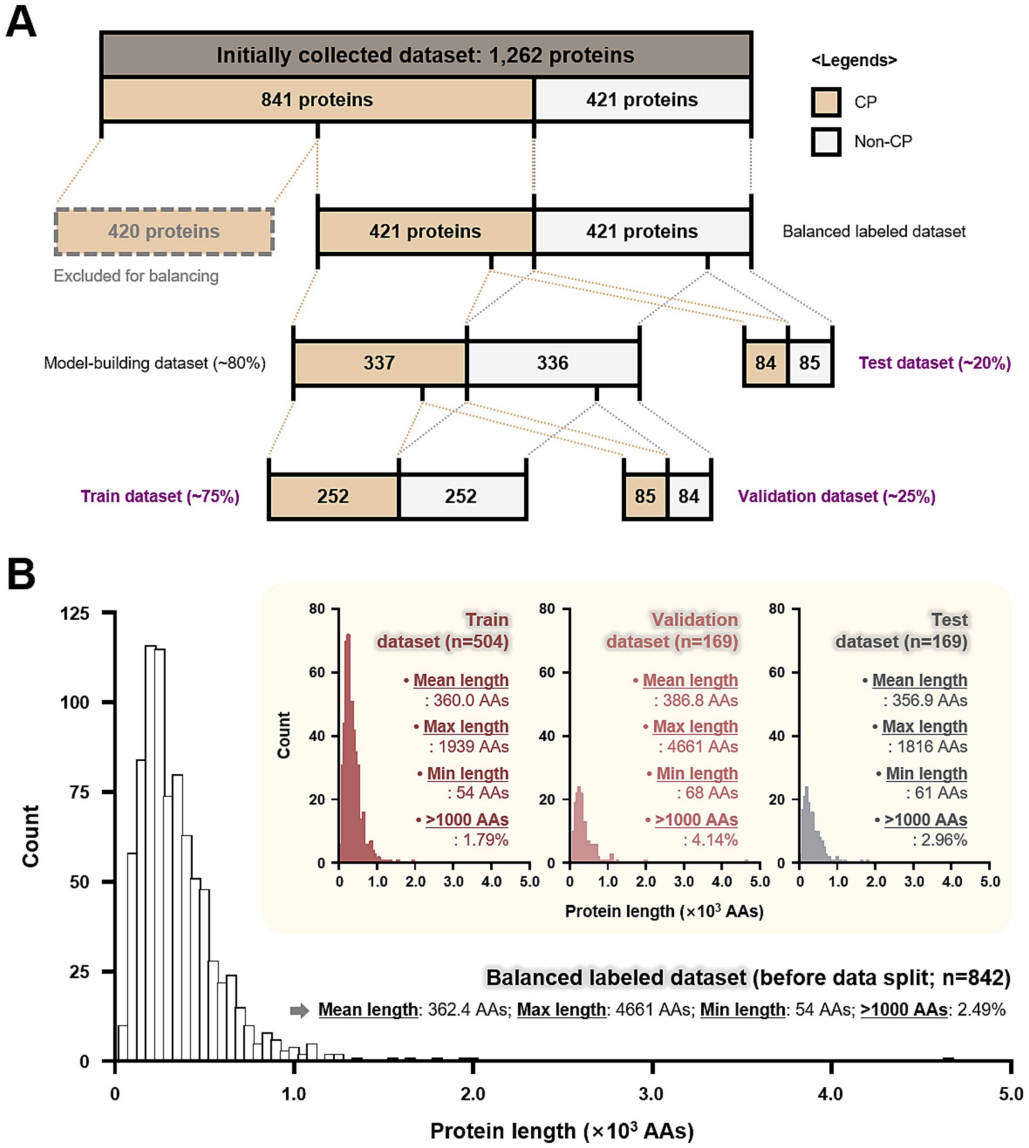
Composition and distribution of the data used to develop Chlamy_ChloroPred. **(A)** Sequences from 1,262 proteins were initially collected, but 420 CP-labeled proteins were excluded to balance the labeling of the data. Through sequential data splitting, train, validation, and test datasets were prepared at a ratio of 3:1:1. The indicated percentages correspond to the proportion of a given dataset compared to the previous one immediately before the split. CP stands for chloroplast protein. **(B)** Histograms demonstrating the length distribution of protein sequences from the balanced labeled dataset, train, validation, and test datasets. Several statistics are shown for each dataset, including the mean, maximum, and minimum length of the protein sequences included. The proportions of proteins with lengths greater than 1,000 amino acids (>1,000 AAs) are also presented. Max and Min stand for maximum and minimum, respectively.

Since we aimed at developing a CP classifier tailored for *C. reinhardtii*, we collected 1,262 protein sequences, of which 841 were revealed to be localized in the chloroplast (i.e., CP) and 421 were revealed to be localized in organelles other than the chloroplast (i.e., Non-CP) (Figure 1A; Supplementary Dataset S1). To furnish the developing program with data whose localizations are empirically corroborated, we manually mined gene identifications from 6 literature sources, including: 5 reports that experimentally investigated the proteomics of the model green microalga using fluorescence-tagged proteins (Wang et al., 2023) and mass spectrometry identifying semi-tryptic peptides (Terashima et al., 2010; Bienvenut et al., 2011; Zhan et al., 2018; Ramundo et al., 2020); and one reference that thoroughly scrutinized the subcellular destinations of proteins reported in various publications using its own stringent criteria (Tardif et al., 2012). Protein sequences corresponding to the gene identifications were then scrapped from the *Chlamydomonas reinhardtii* v5.6 database on the Phytozome (Merchant et al., 2007). Only primary transcripts were used for building this dataset. During the compilation of amino acid sequences from the references, redundant sequences (i.e., the identical sequence found in multiple sources) were treated as follows: (i) Sequences designated with the same localization were merged (i.e., one was selected); and (ii) those with controversial localizations were removed to avoid any reasonable doubt. After assembly, 420 CPs were excluded from the overall dataset to balance the data distribution. This resulted in the “balanced labeled dataset” consisting of an equal number of CPs and Non-CPs (421 each; Figure 1A), given that imbalanced input data may induce systematic bias in the model (Belhaouari et al., 2024). Subsequently, we split the resulting dataset into “train (Supplementary Dataset S2),” “validation (Supplementary Dataset S3),” and “test (Supplementary Dataset S4)” datasets with a ratio of 3:1:1, while maintaining the label distribution of each subset (Figure 1B).

### 2.2 Neural network model

**Figure 2:**
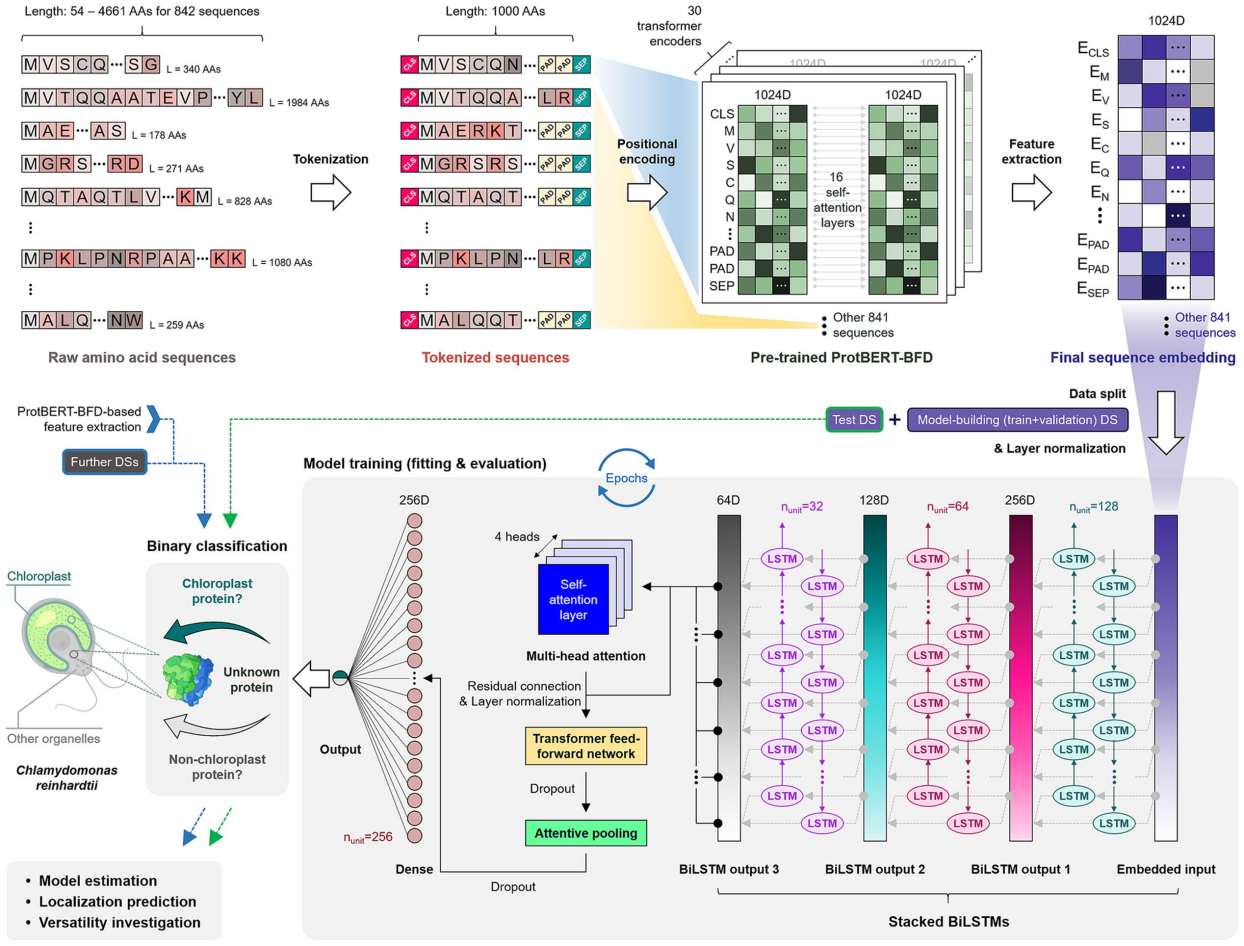
Schematic diagram describing the architecture and binary prediction process of Chlamy_ChloroPred. Raw amino acid sequences, which are composed of single-letter amino acid codes, were processed for length normalization to 1,000 AAs and tokenized using the CLS (classification token), PAD (padding token), and SEP (separator token). A large protein language model, ProtBERT-BFD, was employed to encode the input protein sequences with pre-learned biochemical and structural features in a form suitable for the subsequent prediction layer. The resulting embeddings were input into an iterative prediction network consisting mainly of a stacked RNN-BiLSTM (recurrent neural network-bidirectional long short-term memory) and an attentive pooling layers. The training resulted in the establishment of the binary chloroplast protein classifier, Chlamy_ChloroPred, which was then used to estimate performance, predict protein prediction, and investigate versatility. Further DSs refer to additional datasets (DSs) that could be applied to the model (e.g., the *A. thaliana* proteome) for broader applications, other than the train, validation, and test datasets.

We designed a deep learning-based classifier framework that integrates a stacked recurrent neural network and attention-based mechanisms to forecast CPs from ProtBERT-BFD embeddings (Figure 2). Prior to the model organization, the datasets were labeled in a binary fashion as 0 and 1 for Non-CPs and CPs, respectively, to proceed with supervised learning. After labeling, all protein sequences subjected to the train, validation, and test processes were fixed at a maximum length of 1,000 amino acids (AAs) from the N-terminus, with longer sequences truncated and shorter ones padded. The standard length of the N-terminal sequences likely containing cTPs (i.e., 1,000 AAs) was determined by considering that cTPs are typically no longer than this limitation (Tardif et al., 2012) and that >95% of sequences in the overall dataset fall within 1,000-AA coverage (Figure 1B), making the model framework lightweight while covering the entire length of the most sequences.

As an initial step in constructing the framework, a pre-trained ProtBERT-BFD model was used to obtain embeddings (i.e., 1,024-dimensional vectors) from datasets, enriched with biochemical and structural context pre-learned from large protein corpora. We used the token-level embeddings derived from the ProtBERT-BFD-based feature extractor as inputs of downstream. A positional encoding layer was applied to the input to preserve positional information lost during embedding. Using a layer normalization, the position-encoded sequences were then normalized. Subsequently, the normalized outputs were processed by a stack of triple bidirectional LSTM (BiLSTM) layers, which retain hidden dimensions of 128, 64, and 32 units, respectively, in order to capture contextual dependencies along the protein sequence in both the forward and backward directions. Next, we added a multi-head self-attention mechanism with 4 heads and 64 key dimensions to the framework to improve the representation with long-range inter-residue context-aware dependencies. The resulting output was incorporated back into the BiLSTM through a residual connection, followed by layer normalization and a Transformer feed-forward subnetwork. This attention block enables the model to dynamically weight sequence positions and understand the relationships (i.e., global dependencies) among all tokens in parallel. Following the contextualization step, we applied another sublayer of the attention mechanism, an attentive pooling layer, which calculates a weighted average of sequence features. This effectively prioritizes biologically informative sequence regions (e.g., cTP) and simultaneously attenuates less relevant residues for classification. Finally, the pooled representation was regularized using dropout with a probability of 0.45 and then passed through a fully connected dense layer of 256 units with rectified linear unit (ReLu) activation and L2 kernel regularization (*λ* = 1 × 10^−4^), followed by the final dropout step consisting of a single unit with a sigmoid activation function that prints the probability of the sequence being localized to the chloroplast. For binary classification, a probability of ≥0.5 was used as the threshold for determining localization.

### 2.3 Program execution

We used Google Colaboratory (Colab) environment (Python 3, T4 GPU, and high-RAM mode) for running the deep learning-based program. The model was trained using the Adam optimizer with a learning rate of 1 × 10^−4^, minimizing the binary cross-entropy loss with prediction accuracy as the evaluation metric. The early stopping callback was used to prevent the neural network from overtraining by monitoring the validation loss with a patience of 3. We saved the best version of the trained model with the best validation accuracy for further evaluation using the model checkpoint callback.

### 2.4 Performance measures of the models

When evaluating the performance of models developed in this study based on the input data described in Section 2.1, the performance metrics including the accuracy, precision, recall—also known as sensitivity or true positive rate (TPR), and F1 score were calculated and compared as follows (Yue et al., 2022):


$$\text{Accuracy}=\frac{\mathrm{TP}+\mathrm{TN}}{\mathrm{TP}+\mathrm{FN}+\mathrm{FP}+\mathrm{TN}}$$ (1)

$$\text{Precision}=\frac{\mathrm{TP}}{\mathrm{TP}+\mathrm{FP}}$$ (2)

$$\text{Recall}\;(\text{sensitivity or}\;\mathrm{TPR})=\frac{\mathrm{TP}}{\mathrm{TP}+\mathrm{FN}}$$ (3)

$$F1\;\text{score}=\frac{2\times \text{Precision}\times \text{Recall}}{\text{Precision}+\text{Recall}}$$ (4)

Where TP is true positive (counted if an instance of CP is correctly predicted as CP), TN is true negative (counted if an instance of Non-CP is correctly predicted as Non-CP), FP is false positive (counted if an instance of Non-CP is wrongly predicted as CP), and FN is false negative (counted if an instance of CP is wrongly predicted as Non-CP). Accuracy measures the percentage of correct predictions across all samples, while precision calculates the proportion of positive classifications that were actually correct and improves as the number of false positive decreases. Recall is defined as the proportion of actual positives that were correctly classified as such and improves when the number of false negative decreases. F1 score is the harmonic mean of precision and recall, which balances the importance of both, indicating the reliability of a model.

Meanwhile, to evaluate the model’s performance (i.e., effectiveness of the binary classification model), we plotted the ROC (receiver operating characteristic) curve and estimated the AUC (area under the ROC curve). The ROC curve visually represents the performance of a model that is graphed by calculating the TPR (Equation 3) against the false positive rate (FPR) at various threshold values. The FPR, which measures how often a model incorrectly predicts negative samples as positive, is calculated as follows (Adabor et al., 2025):


$$\text{False positive rate}\;(\mathrm{FPR})=\frac{\mathrm{FP}}{\mathrm{FP}+\mathrm{TN}}$$ (5)

The AUC is one of the most useful single indicators for comparing the overall performance of models, particularly when the dataset is balanced. An AUC score of 1 means the model achieves perfect classification.

As one of the performance measurements of the developed model, the entire *C. reinhardtii* proteome (i.e., 19,526 proteins provided in *Chlamydomonas reinhardtii* genome version 5.6 in the Phytozome database) (Merchant et al., 2007) was also prepared and tested as described above.

### 2.5 Comparison with benchmark programs

The performance of the proposed model was compared with that of the TargetP 2.0 software (Almagro Armenteros et al., 2019), which was selected as the primary benchmark in this study due to its ease of access, multi-species and cross-proteome versatility, and established authority in the field of protein localization prediction. When running the software, two parameters were selected: “Plant” for the organism group and “Long output” for the output format. Input dataset for the benchmark was identically pre-conditioned as described in Sections 2.1 and 2.2. For the TargetP 2.0 results, a protein was designated as a chloroplast protein when the “Chloroplast transfer peptide” score was the highest among other scores.

To more comprehensively position Chlamy_ChloroPred among currently available models, we examined the predicted localization of proteins in the test dataset (Supplementary Dataset S4) using broader predictors, including TargetP 1.1 (the previous version of TargetP 2.0) (Almagro Armenteros et al., 2019), PredAlgo (Tardif et al., 2012) (based on the prediction results publicly archived in the Phytozome *Chlamydomonas reinhardtii* v5.6 database, as real-time, up-to-date services for both tools are not currently available, as confirmed by server status checks and personnel communication), and PB-Chlamy (Wang et al., 2023)—a SOTA model capable of predicting multiple subcellular localizations, such as mitochondrial, secretory, and other cellular compartments (integratively classified as “other”), in addition to chloroplast localization, as well as assigning potential mitochondrial/chloroplast and secretory/chloroplast dual-targeted protein candidates. Given its ability to distinguish dual-targeted proteins, PB-Chlamy was further used to extract putative dual-targeted protein candidates (Supplementary Dataset S5).

### 2.6 Versatility evaluation of the Chlamy_ChloroPred

The versatility of the Chlamy_ChloroPred program was evaluated using a chloroplast proteome dataset from the model terrestrial plant, *A. thaliana*. The chloroplast protein dataset was basically obtained from the report by Ferro et al. (2010). Of the 1,323 protein identifications (IDs) listed in the study, the amino acid sequences of 1,263 protein IDs (Supplementary Dataset S6), which are now available on the TAIR (The Arabidopsis Information Resource) portal were tested (Reiser et al., 2017). The identical dataset was also applied to the primary benchmark in this study, TargetP 2.0, and the resulting predictions were compared. For the predicted results from TargetP 2.0, proteins that were predicted to be localized in other organelles (e.g., the mitochondria or the extracellular region) were treated as non-CPs. Those that were predicted to be localized in the chloroplast or the thylakoid lumen—as a component of the chloroplast—were treated as CPs.

## 3 Results

### 3.1 Building input dataset

Given the heavy dependence of model performance on the quality of the input data, the preparation of refined input data is imperative in implementing a high-performance predictive program. Considering this, we first balanced the initially collected dataset consisting of 1,262 proteins that include 841 CP-labeled and 421 non-CP-labeled proteins by excluding 420 CP-labeled proteins, leading to a balanced labeled dataset with 421 CP-labeled and 421 non-CP-labeled protein sequences (842 proteins in total). Subsequently, approximately 80% (673 proteins) of the balanced labeled dataset was grouped into the model-building dataset while *ca*. 20% (169 proteins) of the dataset was grouped into the test dataset. The model-building dataset was further split into train and validation datasets, which contain about 75% (504 proteins) and 25% (169 proteins) of the original dataset, respectively. All splitting and grouping processes were conducted while maintaining balance in label distributions (i.e., CP *vs*. non-CP) because it reduces bias toward the majority class, ensures fair training, and improves the performance and interpretability of a binary classification model (Friedberg et al., 2013). As a result, we successfully secured the total dataset, consisting of train, validation, and test subsets in a 3:1:1 ratio (Figure 1A).

Next, we examined the length distribution of the datasets to confirm that they all have a similar dispersion of amino acid lengths. Similar length distributions were observed in the histograms of each split dataset, and it was found that the subsets demonstrate analogous summary statistic values (e.g., mean and minimum lengths) (Figure 1B). On the contrary, the maximum lengths of each subset are disparate because the total dataset (i.e., the balanced labeled dataset) contains a sparse number of proteins over 1,000 AAs (2.49%)—only 21 out of 842 proteins. The maximum length of sequence data in a dataset is of interest and importance since it would establish a standard for length normalization, which is used to uniform input dimensions. Given that using a long input length (e.g., 4,661 AAs in this study) can lead to a high computational burden and rather adversely affect the model’s performance, the input length was standardized to 1,000 AAs, which has a high chance of containing the localization determinant sequence, cTP, without sacrificing the sequence information of more than 95% of protein sequences in any dataset that are shorter than this coverage length (Figure 1B).

### 3.2 Prediction model construction

As shown in Figure 2, a predictive model, named Chlamy_ChloroPred, was constructed in this study that is primarily based on a triple stack of RNN-BiLSTM and an attentive pooling layer. Prior to data input, the length-normalized input sequences were tokenized using special tokens, such as the classification token (CLS), the padding token (PAD), and the separation token (SEP), and then embedded by the ProtBERT-BFD protein language model (Elnaggar et al., 2022), which provides enriched biochemical and structural features pre-trained on a large number of protein sequences. As a result, this model outputs the probability of a protein sequence being localized to the chloroplast with a threshold of 0.5, which implements binary classification (Lee et al., 2022). The detailed parameters are presented in the Materials and Methods section.

### 3.3 Performance evaluation

**Figure 3:**
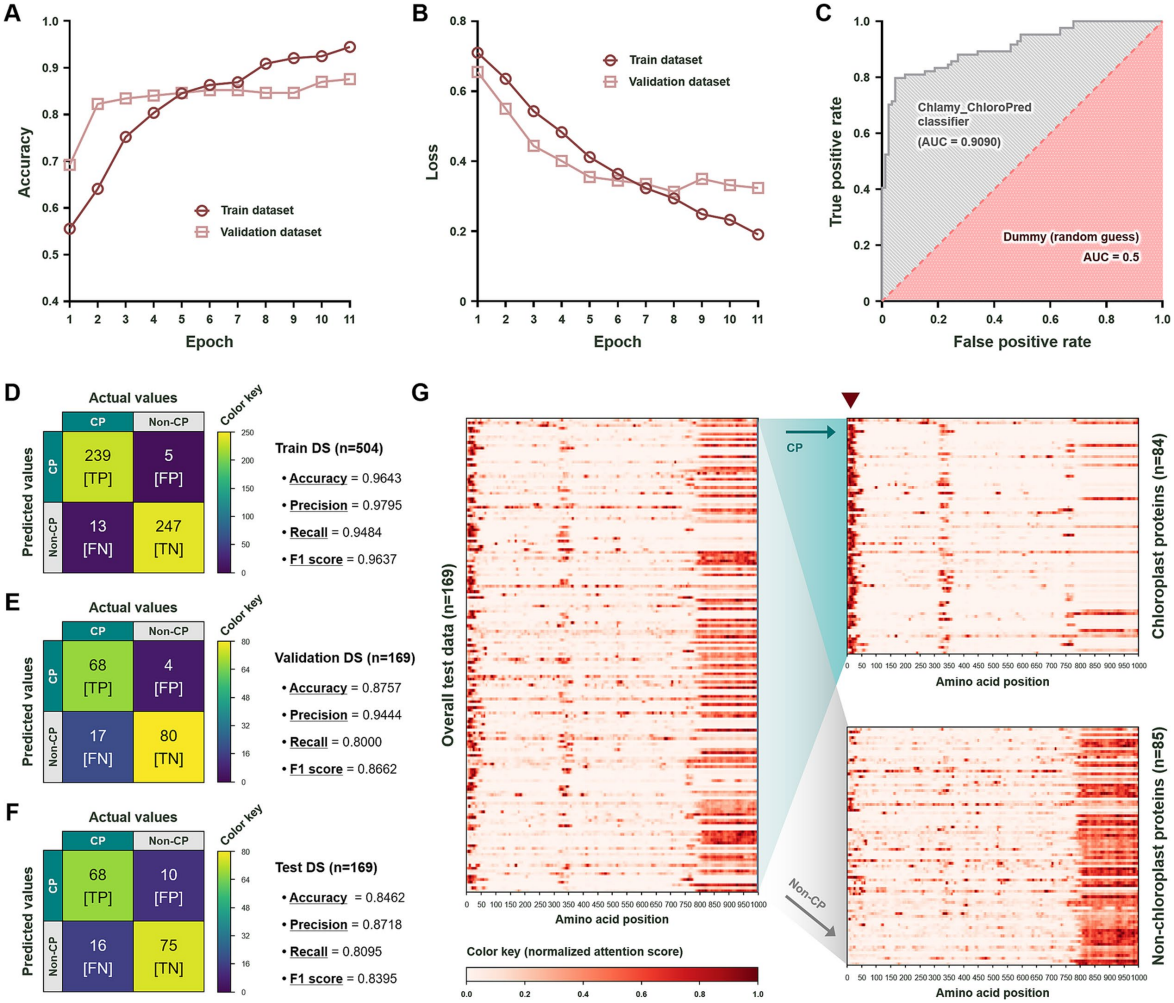
Performance evaluation of Chlamy_ChloroPred and biological interpretation of results from the attentive pooling layer. **(A)** Accuracy and **(B)** loss values plotted against iteration (i.e., epoch) for the train and validation datasets. **(C)** The ROC (receiver operating characteristic) curve for Chlamy_ChloroPred is shown. The AUC (area under the curve) is presented and juxtaposed with that of a dummy classifier. Confusion matrices of the **(D)** train, **(E)** validation, and **(F)** test datasets (DSs). For each dataset, several summary statistic values (i.e., accuracy, precision, recall, and F1 score) are presented. TP, FP, FN, and TN stand for true positive, false positive, false negative, and true negative, respectively. **(G)** Heatmaps showing residue importance during the decision-making process of Chlamy_ChloroPred for the test data. The attention density plot was first illustrated for the overall test data (left panel), then split and separately displayed according to the labels (right panels). Plots of CP and non-CP-labeled proteins are shown in the upper right and lower right panels, respectively. The arrow marks the region to which Chlamy_ChloroPred paid close attention when performing binary classification of CP-labeled proteins.

Under the Google Colab environment, the constructed code was executed. Running was terminated at epoch 11 to prevent overfitting, triggered by the early stopping callback. Since the loss value measures how much the model’s predictions deviate from the true labels, epoch 11 seemed adequate as the stopping point. This is evident from the result that the stopping point occurred before the train and validation losses largely diverged, while the train and validation accuracies increased steadily (Figures 3A,B). Based on the trained program, we graphed the ROC curve and calculated the AUC (Figure 3C) to examine the overall performance of the model. Our classifier showed an AUC of 0.9090, surpassing the 0.5 AUC of a random guess model. This demonstrates that our model is much better than a dummy classifier at distinguishing between positive (i.e., CP) and negative (i.e., non-CP) classes.

More specifically, the model’s inference performance was evaluated with regard to accuracy, precision, recall, and F1 score using the train, validation, and test datasets. The results were presented in the form of confusion matrices (Figures 3D–F). The accuracy (Equation 1), precision (Equation 2), recall (Equation 3), and F1 score (Equation 4) were not lower than 0.8462 (with a loss of 0.4052), 0.8718, 0.8000, and 0.8395, respectively, most of which were derived from the test dataset. This reveals the model’s balanced and reliable performance in predicting whether given proteins in *C. reinhardtii* are localized to the chloroplast or not.

Subsequently, we illustrated heatmaps of residue importance derived from the attentive pooling layer, which was employed to improve the model’s predictive accuracy by focusing attention on relevant sequence features and to offer interpretability through attention weight visualization. As shown in Figure 3G, our model focused selectively on the N-terminal regions of most CP-labeled proteins in the test dataset (upper right panel of Figure 3G), while paying relatively higher attention to the C-terminal regions—comparatively less related to chloroplast localization (Tardif et al., 2012)—of most non-CP-labeled proteins in the same dataset (lower right panel of Figure 3G). Concentrated attention at the *N*-terminal region of non-CPs likely reflects the presence of alternative targeting signals or the absence of canonical chloroplast transit peptide features, thereby providing strong negative evidence for chloroplast localization in a binary classification setting. Meanwhile, the minor attention peaks outside the N-terminal region of CPs are likely attributable to the internal sequence features correlated with CPs (Caspari, 2022) and background noise effects, rather than *bona fide* targeting signals. It is also noteworthy is that the approximate length of the sequences focused on in the CP-labeled proteins corresponds to the previously known average length of cTPs in *C. reinhardtii* (~50 AAs; arrowed in Figure 3G) (Tardif et al., 2012), strongly implying that, as intended, our model pays close attention to the *N*-terminal extensions (i.e., cTP regions) rather than treating every amino acid region equally when predicting the localization of putative chloroplast proteins.

### 3.4 Performance comparison with benchmark programs

**Figure 4:**
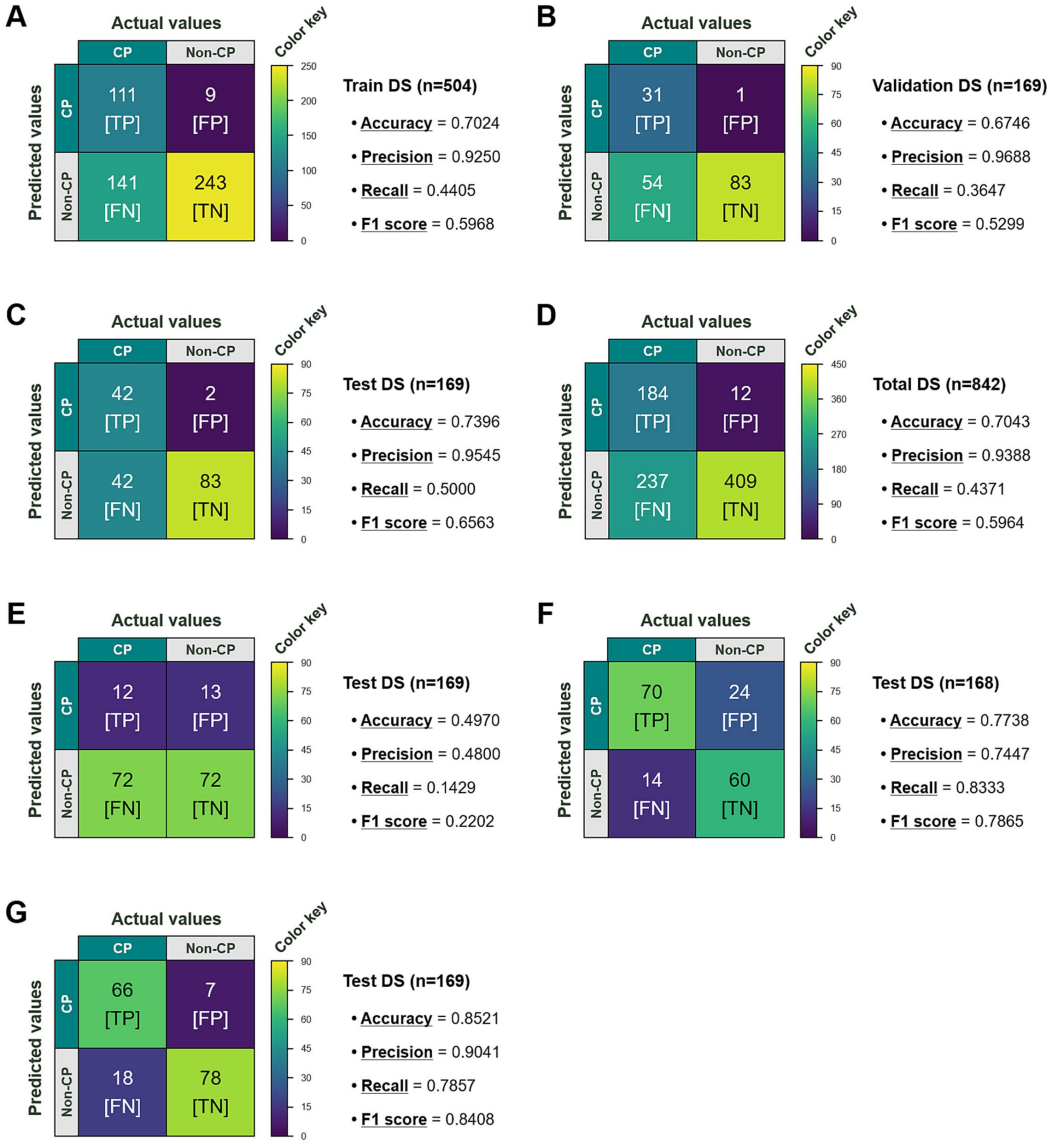
Prediction performance of the primary benchmark in this study, TargetP 2.0, was evaluated in terms of accuracy, precision, recall, and F1 score individually for the **(A)** train, **(B)** validation, and **(C)** test datasets (DSs), as well as for the **(D)** total dataset (i.e., the balanced labeled dataset in Figure 1A), enabling direct comparison with the performance of our developed program, Chlamy_ChloroPred. For broader and more comprehensive comparison, predictions using **(E)** TargetP 1.1, **(F)** PredAlgo, and **(G)** PB-Chlamy were also performed on the identical test DS. It should be noted that, because predicted localization information from the PredAlgo predictor for one protein (Cre14.g634279) is missing from the Phytozome database (Supplementary Dataset S4), the associated statistical analyses were conducted using a total of 168 proteins (*n* = 168). TP, FP, FN, and TN stand for true positive, false positive, false negative, and true negative, respectively.

To objectively validate the merit of Chlamy_ChloroPred, we evaluated the summary statistic values (e.g., accuracy, precision, recall, and F1 score) of the primary benchmark protein localization predictor, TargetP 2.0, using the train (*n* = 504; Figure 4A), validation (*n* = 169; Figure 4B), test (*n* = 169; Figure 4C), and total (*n* = 842; Figure 4D)—the aggregate total of the split datasets, which is the same as the balanced labeled dataset in Figure 1A—datasets in this study and then collated the performance indices of our model (Figures 3D–F) with those of TargetP 2.0. By and large, TargetP 2.0 exhibited higher precision values, but lower accuracy and recall values, across the datasets. Despite the higher precision values, the much lower recall values contributed to the inferior F1 scores of TargetP 2.0. The precision-recall trade-offs observed from TargetP 2.0—i.e., prioritizing precision over recall—implicate that the benchmark was likely programmed with strict, conservative classification criteria for chloroplast proteins. Given the increased accuracy (from 0.7396% to 0.8462%, a 14.41% improvement) and F1 score (from 0.6563% to 0.8395%, a 27.91% improvement) observed on the test dataset (Figures 3F,C), it is evident that Chlamy_ChloroPred outperforms the existing benchmark. To more comprehensively establish the position of our developed model among broader predictors, we further examined the predicted localizations of proteins in the test dataset (Supplementary Dataset S4) using TargetP 1.1 (Figure 4E), PredAlgo (Figure 4F), and PB-Chlamy (Figure 4G). As a result, Chlamy_ChloroPred demonstrated superior classification performance compared with the legacy predictors, including TargetP 1.1 and PredAlgo, in terms of accuracy (70.26% and 9.36% improvements, respectively), precision (81.63% and 17.07% improvements, respectively), and F1 score (281.24% and 6.74% improvements, respectively). When compared with the current SOTA model, PB-Chlamy, Chlamy_ChloroPred exhibited comparable and competitive binary classification performance, showing a modest improvement in recall (3.03%), accompanied by marginal reductions in accuracy (0.69%), precision (3.57%), and F1 score (0.15%). Collectively, these results suggest that Chlamy_ChloroPred represents a viable alternative to existing models, particularly in cases where reliable binary chloroplast protein classification in *C. reinhardtii* is required.

### 3.5 Versatility of Chlamy_ChloroPred

Although our program was constructed as a binary chloroplast protein classifier solely based on the *C. reinhardtii* proteome, we proceeded to use Chlamy_ChloroPred to predict protein localization for the chloroplast proteome of the model terrestrial plant, *A. thaliana*, in order to examine its potential compatibility with other photosynthetic organisms, which could lead to broader applications. In this versatility test, the TargetP 2.0 program was again employed as the comparative benchmark, given its validated multi-species and cross-proteome versatility. For the publicly available *A. thaliana* chloroplast proteome collected from the previous report and the TAIR database (Ferro et al., 2010; Reiser et al., 2017), Chlamy_ChloroPred classified 924 proteins as CPs out of 1,263 CP-labeled proteins in *A. thaliana*, whereas TargetP 2.0 predicted only 821 as CPs (Table 1). As a result, the accuracy and F1 score were 0.7316 and 0.8450, respectively, for our model and 0.6500 and 0.7879, respectively, for the benchmark. Thus, improvements of 12.6% and 7.25% were observed in the accuracy and F1 score of Chlamy_ChloroPred, respectively, compared to TargetP 2.0—although the precision and recall values are less meaningful in this evaluation due to the systematic absence of TN and FP cases, since the input dataset is composed entirely of CP-labeled data, which is fully biased. On balance, we confirmed that this model has the potential to be versatile for various photosynthetic organisms with the core photosynthetic organelle, the chloroplast, as evidenced by its superior prediction performance for the *A. thaliana* chloroplast proteome compared to the commonly referenced protein localization predictor for plants, TargetP 2.0 (Björnsdotter et al., 2021; Sanaboyana and Elcock, 2024).

**Table 1:** Versatility of Chlamy_ChloroPred for the *A. thaliana* CP database.

For *A. thaliana* CP database (*n* = 1,263)	Predicted result^*^		Performance metrics (summary statistics)			
	CP	Non-CP	Accuracy	Precision^†^	Recall^‡^	F1 score
Chlamy_ChloroPred	924	339	0.7316	N/A	0.7316	0.8450
TargetP 2.0	821	442	0.6500	N/A	0.6500	0.7879

The summary statistic performance metrics were compared with those from TargetP 2.0 benchmark for the same input dataset. CP stands for chloroplast protein.

^*^For the predicted results from TargetP 2.0, proteins predicted to be localized in other organelles (e.g., the mitochondria or the extracellular region) were treated as non-CPs, while those predicted to be localized in the chloroplast or the thylakoid lumen were treated as CPs.

^†^Precision is not an applicable metric for this specific dataset (i.e., the *A. thaliana* chloroplast proteome), which is entirely biased toward CPs. N/A denotes not applicable.

^‡^According to the Eqs. 1, and 3, since TN and FP are zero, the recall should be the same as the accuracy.

## 4 Discussion

As interest in biological CO_2_ conversion and chloroplasts, core intracellular organelles involved in photosynthesis, has increased, several predictors have been developed that can infer the presence of chloroplast proteins in a given proteomic database, but their performance and reliability remain unsatisfactory. To address this issue, we developed a new predictor, named Chlamy_ChloroPred, constructed based on a refined proteome from *C. reinhardtii*—a photosynthetic microorganism that serves as a model chassis for various molecular biology and genetic engineering studies of the chloroplast. Aided by embeddings enriched with diverse informative features derived from the large protein language model, ProtBERT-BFD, our model—featuring a complex architecture composed of a triply stacked BiLSTM network coupled with an attentive pooling layer—achieved accurate binary classification of chloroplast proteins in the *C. reinhardtii* proteome, attaining a notable test accuracy of 0.8462. This performance is consistent with previous findings showing that the integration of large language models (LLMs) with attention mechanisms represents a promising direction for future model development (Kong et al., 2024a; Kong et al., 2024b). The prediction performance surpasses that of widely used predictors, including TargetP 1.1, TargetP 2.0, and PredAlgo, which achieved accuracies of 0.4970, 0.7396, and 0.7738, respectively, on the same dataset. Furthermore, our results demonstrate that the proposed model serves as a reliable alternative to the current SOTA predictor, PB-Chlamy, exhibiting competitive performance across multiple evaluation metrics.

This outperformance is likely due to our model’s ability to capture a key region (i.e., amino acid residues) that mediates the destination of a chloroplast-localized protein, as evidenced by the following two findings. Firstly, our model demonstrates a high prediction accuracy with a balanced F1 score despite taking only the foremost 1,000 amino acid regions of proteins as inputs, while sacrificing the ensuing residues of proteins longer than 1,000-AA, instead of using the entire sequences—which is devised to keep the program lightweight. Intriguingly, when we tried to execute the program preliminarily with a longer input length normalized to 4,661 AAs, considering the longest sequence in the dataset (Figure 1B), we found that the trial rather reduced the model’s predictive performance and increased the program’s computational demand (data not shown). The observation, truncating the C-terminal regions did not impair prediction performance, suggests that the model effectively identifies and exploits biologically informative residues within the *N*-terminal regions, on which the inference of chloroplast localization is based. Secondly, and more specifically, Chlamy_ChloroPred focused intensively on the most front ~50-AA sequences of chloroplast proteins (Tardif et al., 2012), which are potential locations for cTP, using its locality-awareness conferred by the attentive pooling layer (Figure 3G) when making the final classification determination.

Another advantage of our model is its versatility, which may allow for wider compatibility with other photosynthetic organisms, as exemplified by the investigation using the *Arabidopsis* chloroplast proteome. Achieving an accuracy of 0.7316, Chlamy_ChloroPred demonstrates a 12.6% improvement over the widely used benchmark program TargetP 2.0, which has validated multi-species and cross-proteome versatility, despite being originally developed for the precise binary classification of potential chloroplast proteins in *C. reinhardtii* using only the *Chlamydomonas* proteome database. This may also be attributed to our model’s capability to apprehend the conserved characteristics of CP across organisms—albeit the inexplicitly revealed common features of CP (Bruce, 2001), which is empowered by the well-designed architecture employing the large-scale, pre-learned protein features obtained from ProtBERT-BFD.

Meanwhile, compared with other predictive models, our model exhibits a tendency toward permissive classification, in turn leading to a relatively high number of false positives. For example, when we applied Chlamy_ChloroPred to predict protein localizations for the whole *C. reinhardtii* proteome, it identified 5,335 putative chloroplast proteins out of 19,526 entire proteins, corresponding to 27.32% of the total proteome. The predicted proportion is quite higher than that reported in a previous study (*ca*. 13%) (Wang et al., 2023), suggesting that our model adopts relatively liberal and permissive criteria for inferring protein localization. This is consistent with the higher FPR of 0.1176 observed in our model (Figure 3F) compared to the benchmark of 0.0235 (Figure 4C) for the test dataset in this study, as calculated using Equation 5, implying that Chlamy_ChloroPred can occasionally produce false positive predictions, even considering the conservative classification propensity of TargetP 2.0 (see Section 3.4).

In addition to the use of a strictly balanced dataset—which does not reflect real-world proteome distributions, where non-chloroplast proteins predominate, and may contribute to an increase false positive rate (Pawlicki et al., 2020), several factors may also influence the elevated false positive rate of this model, such as ambiguity between mitochondrial targeting peptides (mTPs) and cTPs, which serve as key determinants of the final protein destinations (Kunze and Berger, 2015). To investigate the impact of this potential factor on our model in greater depth, we evaluated its ability to discriminate between chloroplast and mitochondrial proteins by systemically examining and comparing its predictions with those of other predictors used in this study for genes included in the test dataset (Supplementary Dataset S4). We focused on examining specific false positive cases—namely, instances in which mitochondrial proteins were incorrectly predicted as chloroplast proteins. We initiated this analysis by retrieving mitochondrial proteins with previously experimentally validated localizations (Wang et al., 2023) from the protein set under investigation. This led us to compile a set of 12 mitochondrial proteins (Cre09.g393506, Cre10.g449100, Cre07.g349350, Cre13.g563150, Cre02.g088000, Cre03.g157700, Cre01.g054500, Cre10.g420700, Cre09.g393210, Cre12.g496750, Cre10.g428300, and Cre10.g440400), including three dual-targeted proteins (Cre01.g054500, Cre10.g440400, and Cre12.g496750). Among these, the former two are mitochondrial/chloroplast dual-targeted proteins, whereas the latter is a mitochondrial/cytoplasmic dual-targeted protein. To facilitate an intuitive comparison of predictor-dependent false positive frequencies, we excluded the dual-targeted proteins from the analysis. Under this condition, the resulting false positive frequencies were 0.22 (2/9), 0.33 (3/9), 0.11 (1/9), 0.00 (0/9), and 0.11 (1/9), for Chlamy_ChloroPred, PredAlgo, TargetP 1.1, TargetP 2.0, and PB-Chlamy, respectively (Supplementary Dataset S4). This finding suggests that Chlamy_ChloroPred exhibits a measurable capability to discriminate chloroplast proteins from potentially confounding mitochondrial proteins. However, the sharpness of this discrimination remains limited, reflecting a slightly more permissive behavior relative to predictors with lower false positive rates (FPRs), such as TargetP 2.0 (FPR = 0.0235) (Figure 4C) and PB-Chlamy (FPR = 0.0824) (Figure 4G), which may be associated with the elevated FPR observed for our model.

Withal, such permissiveness may be advantageous for capturing chloroplast protein candidates among dual-targeted proteins, as some predictors frequently miss these candidates due to ambiguity in their N-terminal targeting signals (Gould et al., 2024). This notion is supported by our localization analysis based on 48 putative dual-targeted proteins predicted by PB-Chlamy (Supplementary Dataset S5), which was conducted in consideration of both the scarcity of experimentally validated dual-targeting proteins and the high reported accuracy PB-Chlamy, together with its capability to explicitly annotate dual-targeted proteins. As a result, among the 48 putative dual-targeted candidates, PredAlgo, TargetP 1.1, TargetP 2.0, and Chlamy_ChloroPred predicted 39 (81.25%), 22 (45.83%), 18 (37.50%), and 38 (79.17%) proteins as chloroplast proteins, respectively. In parallel, PredAlgo classified 6 proteins as mitochondrial, whereas TargetP 1.1 and TargetP 2.0 assigned 25 and 14 proteins, respectively, to mitochondrial localization. Meanwhile, Chlamy_ChloroPred predicted the remaining 10 proteins as non-chloroplast proteins. These results provide a clue that our developed model, Chlamy_ChloroPred, a degree of relatively effectively captures chloroplast localization as one of the possible destinations of putative chloroplast-related dual-targeted proteins. At the same time, these findings stress the need for continued model development to enable more complete resolution of dual-localization-related ambiguities, thereby allowing more comprehensive capture of chloroplast proteins within dual-targeted pools.

The current restriction of our model to binary classification of proteins as either CPs or non-CPs represents an additional opportunity for future improvement. In the interim, combinatorial use with other specialized predictors targeting specific subcellular regions, such as the nucleus, mitochondria, and extracellular regions (Brameier et al., 2007; Zhao et al., 2019; Savojardo et al., 2020), could exert a synergistic effect in resolving the localization of proteins whose subcellular destinations remain controversial or experimentally undefined. Moreover, while Chlamy_ChloroPred appears capable of correctly assigning certain subchloroplast-localized proteins to the chloroplast, as illustrated by its classification of a thylakoid lumen protein (Cre06.g256250; Supplementary Dataset S4) as a chloroplast protein (He et al., 2023), its current capability is limited to collapsing distinct subchloroplast localizations into a single chloroplast category, thereby hindering fine-grained localization resolution. Consequently, achieving higher specificity in subchloroplast localization will require extension of the current prediction framework. One key direction is to expand Chlamy_ChloroPred to enable discrimination among subchloroplast compartments, including the envelope, stroma, thylakoid membrane, and thylakoid lumen. Provided that sufficient high-quality, experimentally validated training data with precisely annotated subchloroplast localization become available, such an upgraded model could function as a standalone tool to facilitate in-depth chloroplast research across diverse organisms. Furthermore, although only a few archives that facilitate convenient comparison between predicted and experimentally validated localization data—primarily based on fluorescence-tagged datasets—are currently available (Mackinder et al., 2017; Li et al., 2019; Wang et al., 2023), many comparative analyses still rely heavily on manual retrieval from dispersed references. In this regard, integrating an automated framework into our model to systematically link prediction outputs with a broader range of experimental evidence, including immunohistochemistry data (Bernstein et al., 1994), would substantially enhance the user-friendliness and strengthen prediction reliability.

## 5 Conclusion

We successfully developed a deep learning-based framework, Chlamy_ChloroPred, which integrates meticulously designed neural layers while maintaining low computational demand, achieving high predictive accuracy and interpretability. While exhibiting modest permissiveness in classification, Chlamy_ChloroPred outperforms widely used protein localization predictors, including the TargetP series (versions 1.1 and 2.0) and PredAlgo, in chloroplast protein classification for the proteome of the model microalga, *C. reinhardtii*, and demonstrates competitive performance relative to the current SOTA model, PB-Chlamy, under the same binary classification setting. Furthermore, our model exhibits potential versatility across broader photosynthetic lineages, as exemplified by its applicability to the proteome of the representative terrestrial plant, *A. thaliana*. Given that the chloroplast is a nearly ubiquitous component among primary producers (Surridge, 2022) and plays a crucial role in photosynthesis—the principal biological CO_2_ fixation mechanism, Chlamy_ChloroPred would serve as a powerful computational proxy for cumbersome localization experiments, thereby expediting both fundamental chloroplast research and applied engineering efforts aimed at enhancing the CO_2_ assimilation capacity and crop yield of phototrophic organisms.

## Data Availability

The datasets presented in this study can be found in online repositories. The names of the repository/repositories and accession number(s) can be found in the article/Supplementary material. All scripts and trained models used in this study are publicly available at a GitHub repository (https://github.com/superstitione/Chlamy_ChloroPred).

## Supplementary material

SUPPLEMENTARY DATASET S1 Initially collected dataset (*n*=1262).

SUPPLEMENTARY DATASET S2 Train dataset (*n*=504).

SUPPLEMENTARY DATASET S3 Validation dataset (*n*=169).

SUPPLEMENTARY DATASET S4 Test dataset (*n*=169).

SUPPLEMENTARY DATASET S5 Putative dual-targeted proteins (*n*=48).

SUPPLEMENTARY DATASET S6 Arabidopsis thaliana chloroplast proteins (*n*=1263).

## Glossary

AA: Amino acid
AI: Artificial intelligence
AUC: Area under the ROC curve
CO_2_: Carbon dioxide
CP: Chloroplast protein
cTP: Chloroplast transit peptide
DNA: Deoxyribonucleic acid
FN: False negative
FP: False positive
FPR: False positive rate
mTP: Mitochondrial targeting peptide
ProtBERT-BFD: Protein-based bidirectional encoder representations from transformers-big fantastic database
RNN-BiLSTM: Recurrent neural network-bidirectional long short-term memory
ROC: Receiver operating characteristic
SOTA: State-of-the-art
TAIR: The Arabidopsis information resource
TN: True negative
TOC-TIC: Translocon at the outer chloroplast membrane—translocon at the inner chloroplast membrane
TP: True positive
TPR: True positive rate
